# Folding mutations suppress early beta-cell proliferation

**DOI:** 10.7554/eLife.43475

**Published:** 2018-12-14

**Authors:** Honey Modi, James D Johnson

**Affiliations:** 1 Diabetes Research Group, Life Sciences Institute, Department of Cellular and Physiological SciencesUniversity of British ColumbiaVancouverCanada

**Keywords:** beta-cell proliferation, insulin gene mutations, mTORC1, beta-cell maturation, endoplasmic reticulum stress, diabetes, Human, Mouse

## Abstract

Exploring how proliferation and maturation of beta-cells can be impaired after birth will shed light on the origins of various forms of diabetes.

**Related research article** Balboa D, Saarimäki-Vire J, Borshagovski D, Survila M, Lindholm P, Galli E, Eurola S, Ustinov J, Grym H, Huopio H, Partanen J, Wartiovaara K, Otonkoski T. 2018. Insulin mutations impair beta-cell development in a patient-derived iPSC model of neonatal diabetes. *eLife* **7**:e38519. doi:https://doi.org/10.7554/eLife.38519

**Related research article** Riahi Y, Israeli T, Yeroslaviz R, Chimenez S, Avrahami D, Stolovich-Rain M, Alter I, Sebag M, Polin N, Bernal-Mizrachi E, Dor Y, Cerasi E, Leibowitz G. 2018. Inhibition of mTORC1 by ER stress impairs neonatal β-cell expansion and predisposes to diabetes in the *Akita* mouse. *eLife* **7**:e38472. doi:https://doi.org/10.7554/eLife.38472

Insulin is an essential hormone that is produced by beta-cells in the pancreas, and then released in response to nutrients to regulate metabolism throughout the body. The proliferation rate of beta-cells peaks in the first few months after birth, and then declines throughout childhood to the very low levels seen in adults (Figure 1A; Gregg et al., 2012; Kushner, 2013). Insulin production also ramps up in the neonatal period as beta-cells mature, before steadying over time (Figure 1A; Henquin and Nenquin, 2016). During this post-natal window, it is unclear how stresses associated with insulin production influence the way beta-cells adapt, in number and in functionality, to meet nutritional needs. Knowing more about these mechanisms would improve our understanding of how neonatal diabetes, type 1 diabetes and type 2 diabetes develop.

**Figure 1.**
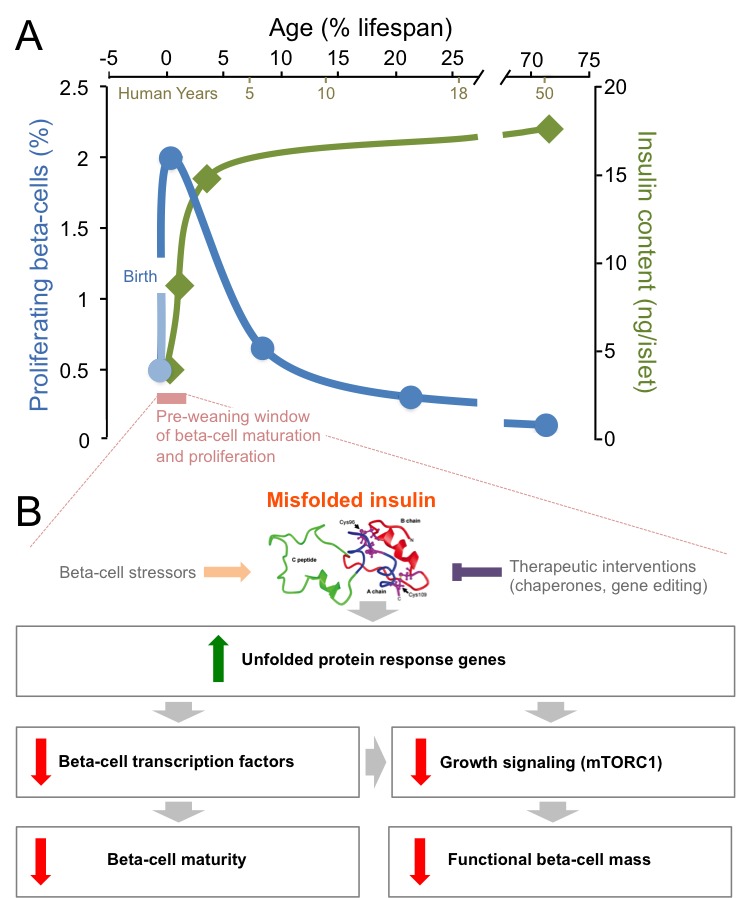
Mutations in insulin and endoplasmic reticulum stress reduce the proliferation and the maturation of beta-cells after birth. (**A**) The post-natal period (pink) is a unique time window when beta-cells proliferate robustly (blue line) and insulin production (green line) increases (Gregg et al., 2012; Henquin and Nenquin, 2016). After that short pre-weaning period, the proliferation rate drops and the production of insulin stabilizes. (**B**) When mutations cause insulin to misfold, the genes in an ER stress pathway known as the unfolded protein response are upregulated (green arrow). These mutations also lead to key beta-cell transcription factors being down-regulated, which impairs the maturation of the beta-cells. Meanwhile, the down-regulation of the mTORC1 pathway also leads to a reduction in the number of functional beta-cells (red arrows). Beta-cell stressors can increase the levels of misfolded insulin (orange arrow), while interventions could therapeutically reduce the levels of misfolded insulin (purple inhibitory arrow).

The functional beta-cell mass is the product of the total amount of beta-cells and their individual responsiveness to nutrients. Functional beta-cell mass is increased by proliferation and maturation and decreased by programmed cell death and the reversion of beta-cells to a less mature state. The synthesis of normal insulin is energetically expensive and inherently stressful, resulting in the suppression of proliferation in normal adult beta-cells (Szabat et al., 2016; Ma et al., 2018). Misfolding of newly synthesized insulin further increases endoplasmic reticulum (ER) stress. In 1997, the Akita mouse strain was reported with a cysteine to tyrosine mutation in the insulin gene at position 96; this mutation results in the protein misfolding, and the animals developing progressive maturity-onset diabetes (Yoshioka et al., 1997). A decade later, it was discovered that a similar mutation in human insulin causes permanent neonatal diabetes (Støy et al., 2007). Mutations of this type account for more than 10% of neonatal diabetes cases, but the molecular mechanisms by which insulin misfolding affects beta-cells were poorly understood. Now, in eLife, researchers in Jerusalem (Riahi et al., 2018) and Helsinki (Balboa et al., 2018) report complementary studies that break new ground in our mechanistic understanding of the effects of insulin mutations on growing and maturing beta-cells.

Gil Leibowitz of the Hadassah-Hebrew University Medical Center and co-workers, including Yael Riahi as first author, conducted a detailed comparison of male Akita and wild-type mice, with a focus on the period just after birth (Riahi et al., 2018). As embryos, there was no difference in how the functional beta-cell mass developed between these animals. However, in pre-weaning mice (19 to 21 days after birth), the beta-cells with mutated insulin were less able to proliferate, which accounted for the shortage of beta-cells found in adult Akita mice. Indeed, there were no changes between the two groups in the rate of programmed death or in the frequency by which beta-cells reverted back to less mature cells. However, as proliferation declines with age, the relative contribution of programmed cell death to beta-cell mass increases in mutant animals (Kushner, 2013; Bachar-Wikstrom et al., 2013).

The Akita mutation was also associated with lower insulin content per beta-cell, as well as diminished insulin release in response to glucose. With these two hallmarks of beta-cell maturity being reduced, the ability to manage blood glucose levels was impaired (Riahi et al., 2018). An unbiased survey of gene expression in newborn mutant animals (prior to high blood sugar levels) showed that beta-cells from Akita mice had elevated markers of ER stress and decreased RNAs that encode for proteins involved in beta-cell maturation. Pathways that regulate cell growth, such as the one that relies on the mTORC1 protein complex, were also negatively affected. Indeed, activating mTORC1 in the beta-cells of Akita mice partially reestablished normal beta-cell mass, insulin content, and control of blood glucose (Figure 1B), but this effect was transient (Riahi et al., 2018). Interestingly, in older Akita mice, inhibiting mTOR was previously shown to improve beta-cell survival and glucose homeostasis (Bachar-Wikstrom et al., 2013). This suggests that the signaling pathway has opposite roles depending on age, and perhaps that it is related to the respective importance of proliferation versus programmed cell death between the pre- and post-weaning stages. Collectively, this detailed analysis of pre-weaning Akita mice revealed that ER stress has an important role in controlling the proliferation and maturation of beta-cells.

To study how mutations in the insulin gene influence human beta-cell maturation, Timo Otonkoski of the University of Helsinki and colleagues – including Diego Balboa as first author – derived ‘stem cells’ from neonatal diabetes patients with mutated insulin, and used them to make beta-like cells that could be grown in the laboratory (Balboa et al., 2018). The team also created a control group of cells with a matching genetic background by using CRISPR-SpCas9 to fix the mutations in the insulin gene. This elegant approach represents the state-of-the-art for determining gene function during human beta-cell development, but better in vitro differentiation protocols will be needed to interrogate the roles of mutant genes in mature cells (Johnson, 2016).

As was observed in the newborn Akita mice, the human beta-like cells with the analogous insulin mutations had increased ER stress, lower rates of proliferation and impaired function compared to the beta-like cells in which the mutations had been fixed. There were no differences in the level of programmed cell death at the developmental stages tested. Surveying the expressed RNAs of individual beta-like cells revealed that, when mutations cause insulin to fold, the genes that encode mTORC1 components, beta-cell transcription factors, mitochondrial proteins, and drivers of proliferation were all reduced. As expected, insulin misfolding resulted in a surge of markers of ER stress (Figure 1B), although it was notable that genes involved in this process were only increased in mature cells that produced high levels of insulin. In fact, it was reported recently that patient-derived beta-like cells that create less insulin, but do not have misfolding mutations, exhibit less ER stress (Ma et al., 2018). This supports the notion that normal insulin synthesis is a significant source of ER stress (Szabat et al., 2016). A recent study suggested that the levels of insulin mRNA can fluctuate 10-fold in individual adult beta-cells (Xin et al., 2018). The mutated cells examined by Balboa et al. showed increased transcription of the insulin gene, yet the levels of the hormone and of its precursor were significantly lower, a phenomenon that deserves to be carefully studied further.

The elegant work of Balboa et al. therefore supports the observations made in diabetic Akita mice, and shows that the relationship between ER stress and beta-cell proliferation also applies during human development. While there are differences between the two species, this robust example of mechanistic conservation takes place in a research climate where some are dismissive of animal models. Both Akita mice and the neonatal diabetes patients from the study by Balboa et al. are born healthy, indicating that the post-natal window is a critical moment where beta-cells are susceptible to ER stress. Many factors other than insulin folding mutations can modulate ER stress and mTORC1 in neonatal beta-cells, including diet. This short period of vulnerability and plasticity after birth may therefore also be important in establishing the risk for type 1 diabetes or type 2 diabetes development later in life.

## References

[bib1] Bachar-Wikstrom E, Wikstrom JD, Ariav Y, Tirosh B, Kaiser N, Cerasi E, Leibowitz G (2013). Stimulation of autophagy improves endoplasmic reticulum stress-induced diabetes. Diabetes.

[bib2] Balboa D, Saarimäki-Vire J, Borshagovski D, Survila M, Lindholm P, Galli E, Eurola S, Ustinov J, Grym H, Huopio H, Partanen J, Wartiovaara K, Otonkoski T (2018). Insulin mutations impair beta-cell development in a patient-derived iPSC model of neonatal diabetes. eLife.

[bib3] Gregg BE, Moore PC, Demozay D, Hall BA, Li M, Husain A, Wright AJ, Atkinson MA, Rhodes CJ (2012). Formation of a human β-cell population within pancreatic islets is set early in life. The Journal of Clinical Endocrinology & Metabolism.

[bib4] Henquin JC, Nenquin M (2016). Dynamics and regulation of insulin secretion in pancreatic islets from normal young children. PLOS ONE.

[bib5] Johnson JD (2016). The quest to make fully functional human pancreatic beta cells from embryonic stem cells: Climbing a mountain in the clouds. Diabetologia.

[bib6] Kushner JA (2013). The role of aging upon β cell turnover. Journal of Clinical Investigation.

[bib7] Ma S, Viola R, Sui L, Cherubini V, Barbetti F, Egli D (2018). β cell replacement after gene editing of a neonatal diabetes-causing mutation at the insulin locus. Stem Cell Reports.

[bib8] Riahi Y, Israeli T, Yeroslaviz R, Chimenez S, Avrahami D, Stolovich-Rain M, Alter I, Sebag M, Polin N, Bernal-Mizrachi E, Dor Y, Cerasi E, Leibowitz G (2018). Inhibition of mTORC1 by ER stress impairs neonatal β-cell expansion and predisposes to diabetes in the *Akita* mouse. eLife.

[bib9] Støy J, Edghill EL, Flanagan SE, Ye H, Paz VP, Pluzhnikov A, Below JE, Hayes MG, Cox NJ, Lipkind GM, Lipton RB, Greeley SA, Patch AM, Ellard S, Steiner DF, Hattersley AT, Philipson LH, Bell GI, Neonatal Diabetes International Collaborative Group (2007). Insulin gene mutations as a cause of permanent neonatal diabetes. PNAS.

[bib10] Szabat M, Page MM, Panzhinskiy E, Skovsø S, Mojibian M, Fernandez-Tajes J, Bruin JE, Bround MJ, Lee JT, Xu EE, Taghizadeh F, O'Dwyer S, van de Bunt M, Moon KM, Sinha S, Han J, Fan Y, Lynn FC, Trucco M, Borchers CH, Foster LJ, Nislow C, Kieffer TJ, Johnson JD (2016). Reduced insulin production relieves endoplasmic reticulum stress and induces β cell proliferation. Cell Metabolism.

[bib11] Xin Y, Dominguez Gutierrez G, Okamoto H, Kim J, Lee AH, Adler C, Ni M, Yancopoulos GD, Murphy AJ, Gromada J (2018). Pseudotime ordering of single human β-cells reveals states of insulin production and unfolded protein response. Diabetes.

[bib12] Yoshioka M, Kayo T, Ikeda T, Koizumi A (1997). A novel locus, *Mody4*, distal to D7Mit189 on chromosome 7 determines early-onset NIDDM in nonobese C57BL/6 (Akita) mutant mice. Diabetes.

